# Challenges in using CellTracker Green on foraminifers that host algal endosymbionts

**DOI:** 10.7717/peerj.5304

**Published:** 2018-08-30

**Authors:** Benjamin J. Ross, Pamela Hallock

**Affiliations:** College of Marine Science, University of South Florida, St. Petersburg, FL, United States of America

**Keywords:** Foraminifera, Epifluorescence, Symbiosis, Dormancy, Mortality, Larger benthic foraminifera, CellTracker Green, Fluorescent probes

## Abstract

The uses of fluorescent microscopy and fluorescent probes, such as the metabolically activated probe CellTracker™  Green CMFDA (CTG), have become common in studies of living Foraminifera. This metabolic requirement, as well as the relatively quick production of the fluorescent reaction products, makes CTG a prime candidate for determining mortality in bioassay and other laboratory experiments. Previous work with the foraminifer *Amphistegina gibbosa*, which hosts diatom endosymbionts, has shown that the species is capable of surviving both acute chemical exposure and extended periods of total darkness by entering a low-activity dormant state. This paper explores the use of CTG and fluorescent microscopy to determine mortality in such experiments, as well as to explore the physiology of dormant foraminifers. The application of CTG was found to be complicated by the autofluorescence of the diatom symbionts, which masks the signal of the CTG, as well as by interactions between CTG and propylene glycol, a chemical of interest known to cause dormancy. These complications necessitated adapting methods from earlier studies using CTG. Here we present observations on CTG fluorescence and autofluorescence in *A. gibbosa* following both chemical exposure and periods of total darkness. While CTG can indicate vital activity in dormant foraminifers, complications include underestimates of total survival and recovery, and falsely indicating dead individuals as live due to rapid microbial colonization. Nonetheless, the brightness of the CTG signal in dormant individuals exposed to propylene glycol supports previously published results of survival patterns in *A. gibbosa*. Observations of CTG fluorescence in individuals kept for extended periods in aphotic conditions indicate uptake of CTG may begin within 30 min of exposure to light, suggesting darkness-induced dormancy and subsequent recovery can occur on short time scales. These results suggest that CTG accurately reflects changes associated with dormancy, and can be useful in laboratory experiments utilizing symbiont-bearing foraminifers.

## Introduction

Fossil foraminiferal shells have been key tools in paleontological applications for more than a century. In the past half century, shell assemblages have also become widely used tools in environmental monitoring and assessment, and live foraminifers are increasingly being used in bioassay applications (i.e., Alve, 1995; Yanko, Arnold & Parker, 1999; Frontalini & Coccioni, 2011). Over the past 20+ years, foraminiferal assemblages and selected populations have become increasingly used to assess and monitor environmental conditions associated with coral reefs, which are in decline worldwide (e.g., De’ath et al., 2012; Perry et al., 2013; others). Hallock (2000) summarized the potential and benefits of using reef-dwelling larger benthic foraminifers (LBF) as indicators of water quality conducive to coral-reef accretion. Experimental approaches have included studies of growth rates (Hallock, Forward & Hansen, 1986), photosynthetic activity (e.g., Talge & Hallock, 2003; Méndez-Ferrer, Hallock & Jones, 2018), prevalence of morphological anomalies (e.g., Prazeres, Uthicke & Pandolfi, 2016), symbiont loss (e.g., Hallock et al., 1995), and, most recently, proteomics (e.g., Prazeres et al., 2011; Stuhr et al., 2018) and antioxidant capacity (i.e., Prazeres, Uthicke & Pandolfi, 2016; Stuhr et al., 2017).

Ross & Hallock (2014) explored the use of an LBF, *Amphistegina gibbosa* d’Orbigny, as a bioassay organism in toxicological studies relevant to coral reefs. During the development of protocols to determine the 48-hr 50% lethal concentration (LC50) of propylene glycol and 2-butoxyethanol (components of dispersants used in the Deepwater Horizon oil spill), we observed that *A. gibbosa* specimens were capable of surviving some level of toxic exposure by entering a dormant state, in which live individuals were visually indistinguishable from dead individuals, showing no signs of activity, which includes extension of the granuloreticulopodia, attachment to substrate, or production of visible waste (see Ross & Hallock, 2016, for further discussion and definition of dormancy in the Foraminifera). The identification of truly dead specimens required the use of a 72-hr recovery period to determine visually which individuals showed no evidence of activity. However, the uncertainty in the resulting LC50 estimates indicated the need for other readily applicable methods for determining mortality. Though seldom considered by researchers, dormancy is widespread among the Foraminifera (see Ross & Hallock, 2016, and references therein). Previous observations of dormancy in *A. gibbosa* under aphotic conditions (Smith & Hallock, 1992) reinforced the need to distinguish dormant from dead individuals and to better understand physiological facets of dormancy.

Fluorescence methods have a long history of use in recognizing live cells in a wide variety of cytological and histological applications (e.g., Johnson et al., 1981; Taylor et al., 1986; Wommack et al., 1992; Patel et al., 2007). The fluorescent probe, CellTracker™ Green CMFDA (CTG), is a non-terminal, non-fluorescent probe that, upon entering a living cell, can be cleaved by non-specific esterases common to living cells, producing a fluorescent compound, fluorescein, visible using a fluorescent microscope (functional mechanism summarized in Bernhard et al., 2006). The requirement of esterase activity means a cell must be alive to produce fluorescence.

How best to distinguish live specimens from dead shells in field samples and experimental treatments is an ongoing challenge and controversy among foraminiferal researchers (i.e., Bernhard et al., 2006; Figueira et al., 2012; Frontalini et al., 2018). Bernhard et al. (2006) compared the effectiveness of CTG with commonly used rose Bengal staining, which stains proteins and can stain dead cytoplasm as well as live. Bernhard and colleagues noted CTG’s effectiveness specifically in identifying live foraminifers with transparent shells, as well as organic-walled (allogromiid) foraminifers. Figueira et al. (2012) similarly showed the benefits of CTG over rose Bengal in identifying live salt-marsh taxa. These studies have generally been concerned with the efficacy of CTG in identifying live specimens from field collections (e.g., by incubating sediment cores).

The use of CTG in foraminiferal laboratory experiments, such as bioassays, is less established. McIntyre-Wressnig et al. (2014) employed it to identify live foraminifers for experiments and Pucci et al. (2009) used it to identify surviving individuals following experimental treatments. The primary objective of our study was to determine if CTG could be used to distinguish live from dead *A. gibbosa* in laboratory experiments. In several previous experimental studies, some specimens that visually appeared to be dead (i.e., normal symbiont color was highly altered and no rhizopodial activity was observed), regained normal golden-brown color and rhizopodial activity after placement in clean seawater with access to light (Smith & Hallock, 1992; McCloskey, 2009; Ross & Hallock, 2014). Our goal was determine to whether CTG could be used to determine activity in dormant foraminifers. The ability of CTG to persist post-fixation (common in methods using CTG on foraminifers, e.g., Bernhard et al., 2006) means that statistically robust numbers of specimens can be experimentally treated, then incubated in CTG, fixed, and observed at a later time. This efficiency, as well as the possibility of automation (e.g., image analysis technology) in place of human observations of vital activity, could greatly increase the potential for applications of *Amphistegina* spp*.* as bioassay organisms.

### Objectives and strategy

The specific goals of this paper were the following: (a) to adapt methods utilizing CTG to observations of *A. gibbosa* in laboratory toxicity experiments; (b) to use these methods to determine if CTG fluorescence is a valid tool for distinguishing mortality versus survival in *A. gibbosa* that may be dormant, including both toxicity- and darkness-induced dormancy; and (c) to determine what fluorescence microscopy can reveal about the activity of dormant individuals.

To achieve these goals, preliminary experiments were required to address the challenge that some substances can interfere with CTG fluorescence. Then four experiments were conducted. Two examined vital activity of *A. gibbosa* exposed to different concentrations of PG for 48 hr. Experiment 1 included a 72-hr recovery period (as in Ross & Hallock, 2014), while Experiment 2 did not. These experiments were conducted to develop a protocol for using CTG in *A. gibbosa* bioassay experiments, and to compare results to determine whether CTG incubation immediately after exposure can indicate vitality in inactive, dormant individuals. Also of interest was whether CTG fluorescence and visually assessed “vital activity” (i.e., extension of granuloreticulopodia, attachment to the sides of well plates, and other visual indicators of life) following the 72-hr recovery period employed by Ross & Hallock (2014) indicated the same survival patterns.

The third experiment assessed vital activity in dormant *A. gibbosa* after 62 days in the dark in a temperature-controlled incubator, based on the results of Smith & Hallock (1992) and BJ Ross & P Hallock (2018, unpublished data) that found *A. gibbosa* can survive at least 20 months in total darkness in an apparently dormant state. The mechanisms of chemically- and dark-induced dormancy, and whether they are functionally the same, are unknown, so determining the effectiveness of CTG fluorescence under different dormancy conditions was of interest. In addition, the techniques developed for Experiments 1 and 2 could be applied without concern for interactions between propylene glycol and CTG.

The fourth experiment was carried out following observations in which high fluorescence was recorded in foraminifers that were visually identified as dead and which exhibited no subsequent recovery. This experiment was performed to determine whether microbial growth in dead *A. gibbosa* could affect the results of fluorescence experiments if individuals died during a 48-hr exposure, and whether this could explain this unexpected fluorescence.

## Methods

Preliminary experiments revealed two challenges in using CTG for these applications. The first challenge was with propylene glycol (PG), the chemical of interest in experiments by Ross & Hallock (2014) that established dose–response curves for *A. gibbosa*. When CTG was added to a PG-seawater treatment medium, no fluorescence was observed either in the media or in the foraminifers, even when PG was present in low concentrations and the foraminifers were active during incubation. This problem was addressed by thoroughly rinsing specimens exposed to PG before placing them in fresh seawater containing CTG and minimizing the length of the CTG incubation. An initial fluorescence presence-absence experiment exposing replicates of five healthy individuals to a range of incubation periods revealed that the shortest incubation period, 30 min, produced visible CTG fluorescence in all individuals; 30 min was thus used as the incubation period in all experiments, including non-PG experiments, for consistency.

**Figure 1:**
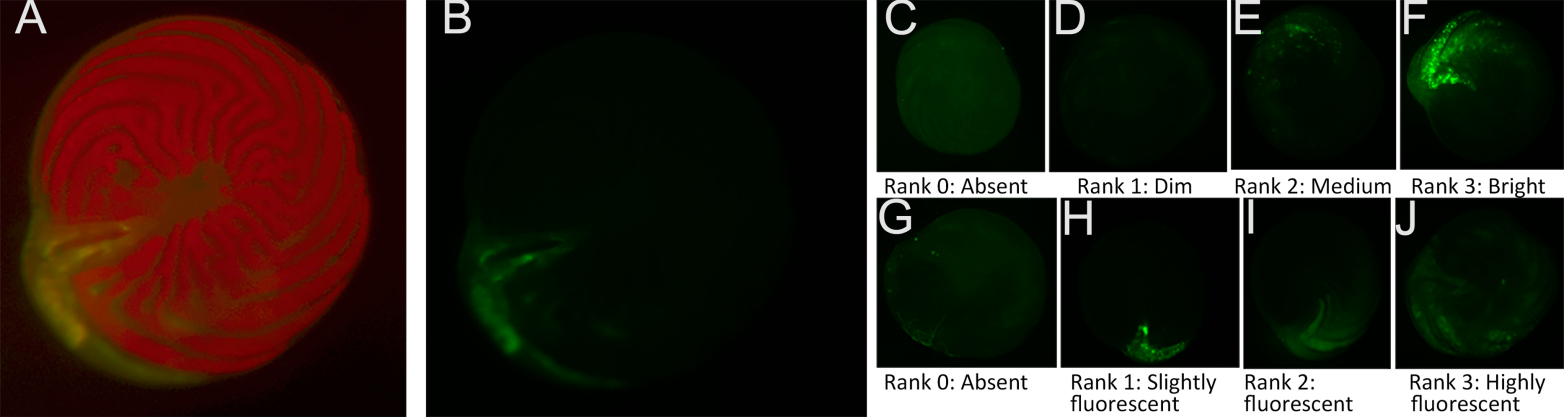
Ranking scale for CellTracker Green fluorescent brightness and coverage in *A. gibbosa*. (A) CellTrackerGreen and photosymbiont autofluorescence vs. (B) isolated CellTrackerGreen fluorescence; (C–F) fluorescence brightness ranking scale; (G–J) fluorescence coverage ranking scale.

The second challenge was because the diatom endosymbionts of *A. gibbosa* exhibit red autofluorescence, which can obscure CTG fluorescence in the endoplasm. As a consequence, CTG fluorescence is most visible in the outer chamber, where the ectoplasm is relatively free of symbionts. As seen in Figs. 1A–1B, even when using filters that exclude the red autofluorescence, CTG fluorescence can primarily be seen in regions of the endoplasm where symbionts are absent.

Challenges associated with autofluorescence were addressed by assessing responses using semi-quantitative ranking of fluorescence brightness and coverage. For fluorescence brightness (Fig. 1B), a ranking of 0 indicates absence of fluorescence; 1 is described as dim fluorescence; 2 as medium fluorescence, easily discerned but not bright; and 3 as bright, often scintillating fluorescence. For fluorescence coverage (Fig. 1C), a ranking of 0 indicates absence of fluorescence; 1 is described as isolated or spot fluorescence, with up to five individual fluorescent spots visible, but not in any obvious distributional relationship; 2 as low coverage (typically in the youngest chamber), with <25% of observable surface area fluorescing green; 3 as medium coverage, between 25% and 70% of the surface area fluorescent; and rank 4 as high coverage, with >70% of the surface area fluorescing green. Rank 1 coverage was generally determined to be the result of contamination or epibiotic growth and for data analysis was combined with rank 0 as representing no fluorescence within the cell.

All experiments were conducted in unfiltered oceanic seawater of salinity ∼38 collected from the sample sites at Tennessee Reef in the Florida Keys (24.7523°, −80.7549–80.7549°), at 25 °C and under a 12-hr dark/12-hr light illumination cycle, with light levels of ∼10 µmol photons/m^2^/s (sufficient for growth and life activity in *A. gibbosa*, without causing photo-oxidative stress; Hallock, Forward & Hansen, 1986; Talge & Hallock, 2003) measured using a LI-COR photometric sensor; the exception to the dark/light cycle was the 62-day darkness treatment.

### Experimental methods

Experiments 1 and 2 applied four experimental treatments, based on results from Ross & Hallock (2014): (a) control concentration (0% PG media); (b) a low concentration of 1.5% (v/v) PG, observed to not cause mortality or initiate dormancy in any foraminifers; (c) an intermediate concentration of 3% PG, observed to cause 100% apparent mortality (lack of rhizopodial activity and waste production) in the experimental specimens following the 48-hr exposure, but exhibiting 100% recovery by 72 hr after removal from PG exposure; and (d) a high concentration of 8% PG, observed to cause 100% apparent mortality and no recovery by 72 hr after removal from PG exposure. In Experiment 2, the high concentration was raised to 10% because a few individuals exposed to 8% PG showed signs of recovery following the first experiment’s 72-hr recovery period. Each experimental treatment included five replicates, and each replicate included five *A. gibbosa* specimens.

For the first PG experiment, the foraminifers were exposed to treatment conditions for 48 hr. Specimens were rinsed with clean seawater three times to remove residual PG, then incubated in 0.3 µM CTG in unfiltered seawater at a salinity ∼38 for 30 min at ∼25 °C. After incubation, the foraminifers were again rinsed three times with clean seawater to remove any remaining CTG, so that uptake would not occur after the 30-min incubation and reflect later recovery. Preliminary experiments revealed that these procedures produced observable CTG fluorescence, while hypothetically limiting recovery of metabolic activity of the foraminifers following removal from a treatment. The foraminifers were then allowed to recover for 72 hr in clean seawater, following the protocols developed by Ross & Hallock (2014). After 72 hr, color and activity of each specimen were visually assessed, then individuals were imaged while living to record presence of fluorescence (as described below in the ‘Imaging and Statistical Analysis’ section).

For Experiment 2, 48-hr duration treatments again were conducted, with the 8% concentration of PG replaced by 10%. In addition, a “dead” control treatment was included to test the hypothesis that dead individuals would exhibit no fluorescence. Specimens were killed by placing the foraminifers in deionized water for the duration of the 48-hr incubation. The foraminifers were rinsed and exposed to CTG using the same procedures as in Experiment 1, but without a 72-hr recovery period. Instead, digital photographs were taken immediately after they were rinsed to remove the CTG. Color and activity of each specimen were visually assessed, then individuals were imaged while living to record the presence of fluorescence.

For Experiment 3, the extended darkness treatments, the foraminifers were kept in replicates of 5 ml microcentrifuge tubes with pinholes pierced at both ends to allow for gas exchange. These tubes were kept in semi-opaque containers, double wrapped in aluminum foil, in a temperature-controlled incubator at 25 °C. After 62 days in darkness, one treatment of five replicates of five specimens each was incubated in CTG for 30 min in darkness. The other was incubated in CTG for 30 min in ambient room light, which is sufficient to allow survival and growth in *A. gibbosa* kept in aquaria with no other light sources. The foraminifers were then photographed live under an epifluorescent microscope.

For Experiment 4, specimens were divided into two treatments of 25 specimens, each placed in seawater in a large, sealed Nalgene bottle, and then killed by exposure to temperatures of 60–65 °C for approximately 4 hr. On removal, the specimens were transferred to well plates filled with seawater for 48 hr before incubation in CTG and imaging. One treatment was kept in seawater from the heat-treated Nalgene throughout the experiment, including during CTG incubation; this seawater was assumed to be relatively sterile. The other treatment received new, untreated seawater after heat treatment. Specimens were individually assessed visually for mortality before and after live fluorescence imaging.

### Imaging and statistical analysis

All images were taken using a Leica MZ FLIII epifluorescent stereomicroscope with FLUOIII filter system. The CTG was excited using a mercury short-arc lamp, and filtered to a range compatible with GFP (green); a Leica Filter cube N3 (Ex546/12 & Em 600/40 with a 565 beam splitter) was used. A standard magnification of 40× was used and the microscope was focused on the foraminifer’s shell using reflected light before fluorescence imaging to standardize focal depth. Standard color images were taken first, then green epifluorescent images (to visualize CTG), followed by combined RGB epifluorescent images (to visualize combined CTG and endosymbiont autofluorescence) using 30 s exposures. Resulting fluorescence was ranked according to scales for both brightness and coverage (Fig. 1). Foraminifers were not fixed, but photographed while living. This was initially done so that they could be observed for recovery of activity following photography. Although they were found to suffer from photic shock post-photography, making this goal unreliable (see Discussion), in later experiments we continued to photograph specimens live and unfixed for consistency across treatments.

Statistical tests were performed using MATLAB with the Fathom toolbox (Jones, 2015). Because distributions were non-normal, all tests performed were non-parametric. Distributions of resulting fluorescence in each experiment were compared using Mann–Whitney tests, single- or two-factor non-parametric Analysis of Variance tests, and non-parametric Analysis of Covariance tests depending on comparisons being made. All tests were considered significant at *p* = 0.05. Error bars in figures represent standard errors of means.

## Results

Of the two response parameters used in this study, fluorescent coverage and fluorescent brightness, the latter was generally more useful. Based on non-parametric ANOVA, significant differences in coverage were only observed in Experiment 1 (72-hr recovery after PG exposure, *df* = 92, *p* = 0.03) between the 1.5% PG and control treatments (*p* = 0.02), 3% PG treatment (0.01) and 8% PG treatment (0.01). No significant difference in coverage was observed in Experiment 2 (assessed immediately after PG exposure, *df* = 96, *p* = 0.6).

Significant differences were seen in fluorescent brightness of specimens among PG treatments in both experiments. In Experiment 1, non-parametric ANOVA showed an overall significant difference (*p* = 0.003) and pairwise comparisons showed differences between the 8% propylene glycol treatment and the control (*p* = 0.02), 1.5% PG (0.001) and 3% PG (0.015). In Experiment 2, a significant difference was observed (*p* = 0.001) between the 3% PG treatment and the control (0.002), 1.5% PG (0.004) and 10% PG (*p* = 0.001) treatments, as well as between the 10% PG treatment and the 1.5% PG treatment (0.004). Although the 10% PG treatment did not differ significantly in brightness or coverage from the control, specimens showed no visual signs of recovery, consistent with previous experiments.

**Figure 2:**
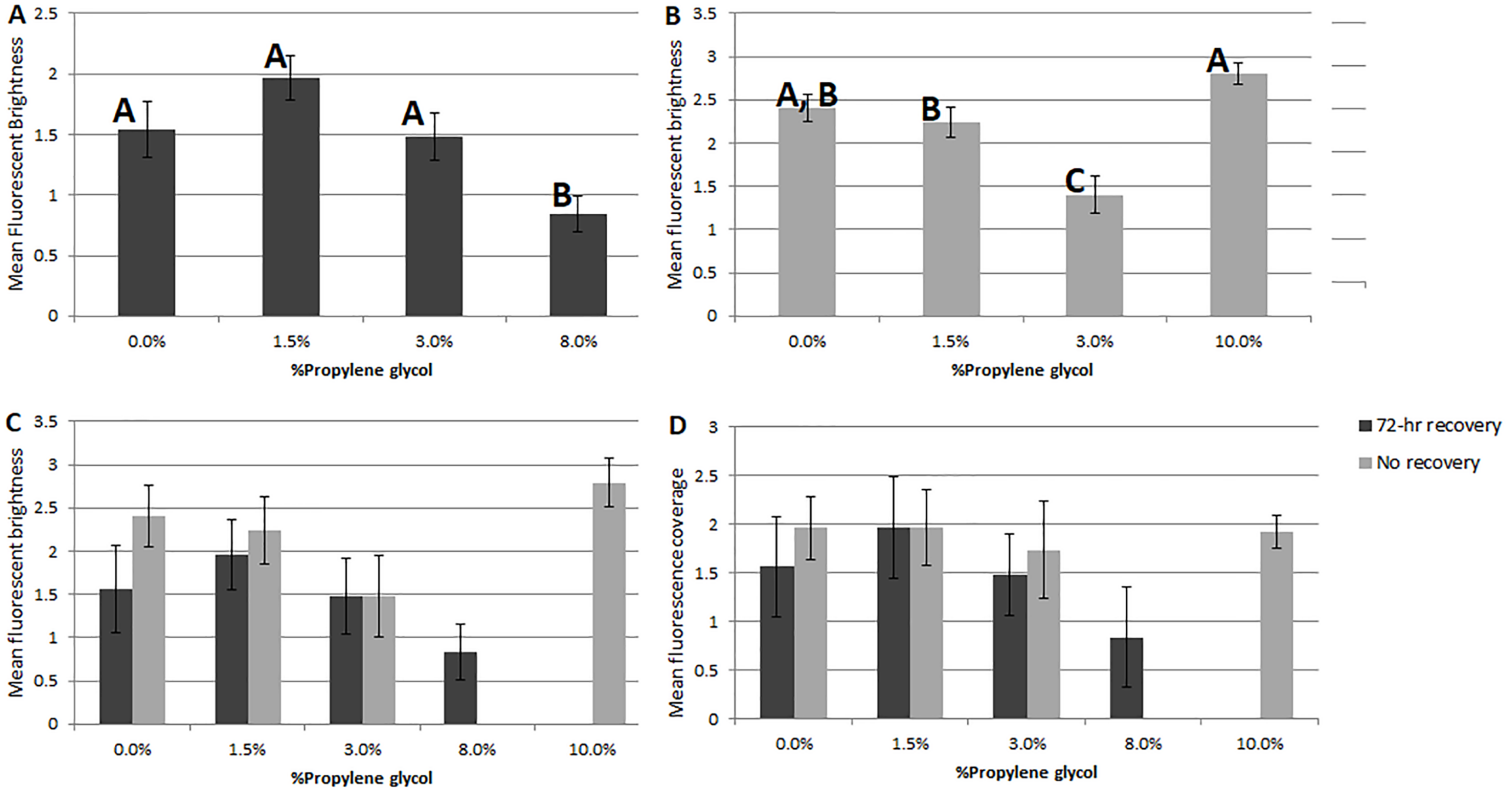
Comparisons of fluorescent brightness and coverage in *A. gibbosa* following 48-hr propylene glycol exposure with and without 72-hr recovery period. (A) Experiment 1–mean rankings in CTG fluorescent brightness following 48-hr propylene glycol exposure and 72-hr recovery in seawater. Letters above the bars indicate that results that are statistically similar; error bars represent standard errors of means; (B) Experiment 2–distribution and significant differences in CTG fluorescent brightness following 48-hr PG exposure with no recovery period; (C) comparison of rankings of fluorescent brightness following 48-hr PG exposure and 72-hr (Exp. 1) or no (Exp. 2) recovery; (D) comparison of rankings of fluorescent coverage following 48-hr PG exposure and 72-hr (Exp. 1) or no (Exp. 2) recovery.

Comparing the two PG-exposure experiments using a 2-factor ANOVA ( *df* = 192), Experiment 2 treatments (no recovery period) showed significantly higher fluorescent brightness (Fig. 2C) and significantly lower coverage (Fig. 2D) than Experiment 1 treatments (factor 1 (recovery vs. no recovery), *p* = 1 × 10^−3^; factor 2 (PG%), *p* = 1 × 10^−3^; factor 1x2, *p* = 1 for both analyses).

**Figure 3:**
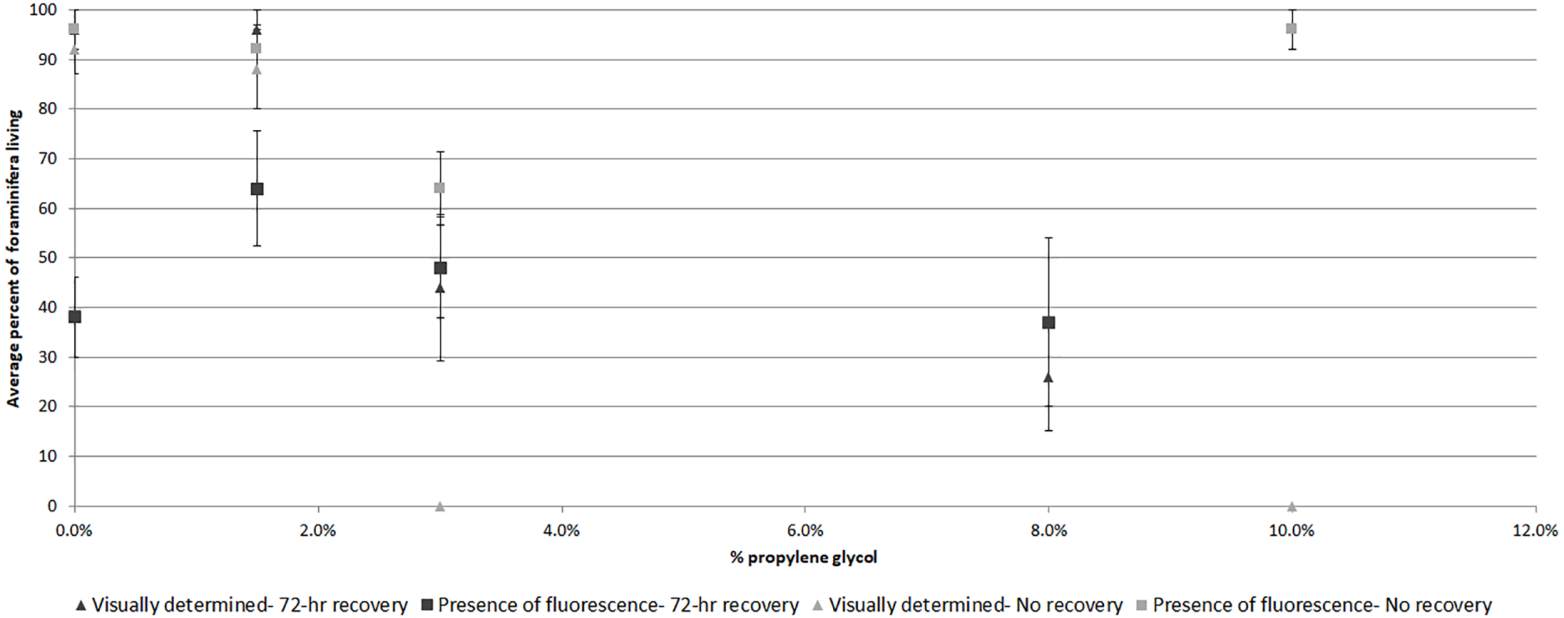
Average percent of foraminifera living after 48-hr exposure to propylene glycol with and without 72-hr recovery period as determined visually and by presence of CTG fluorescence. Average survival as determined by visible signs of activity or by presence of fluorescence following a 72-hr recovery period from PG exposure (Exp. 1) and no recovery from PG exposure (Exp. 2). Error bars represent standard errors of means.

**Figure 4:**
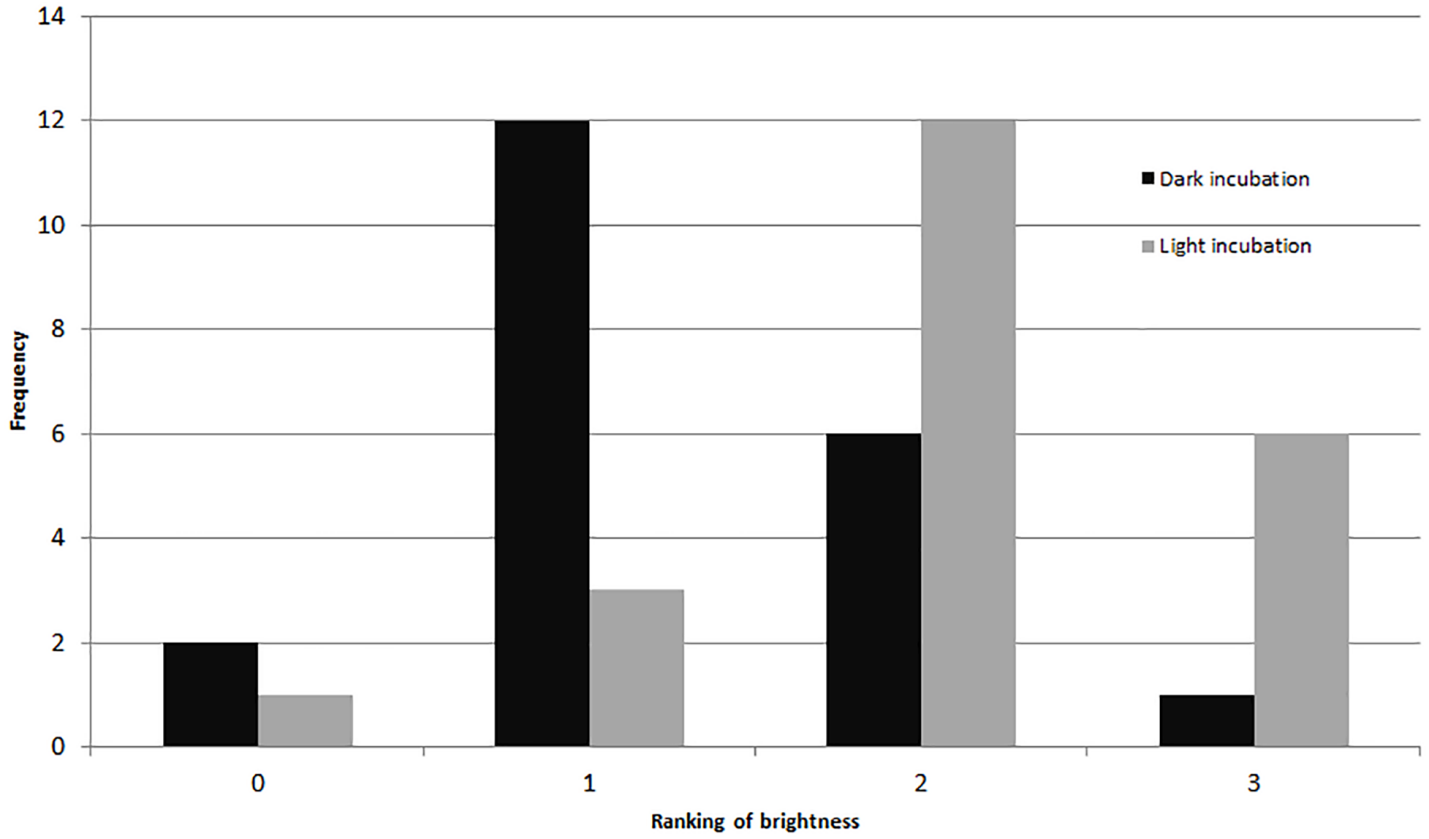
Fluorescent brightness in *A. gibbosa* following 62 days in aphotic conditions. Histogram comparing fluorescent brightness in *A. gibbosa* held in the dark for 62 days, then incubated in CTG in either complete darkness or light (Exp. 3). Frequency includes all specimens observed.

**Figure 5:**
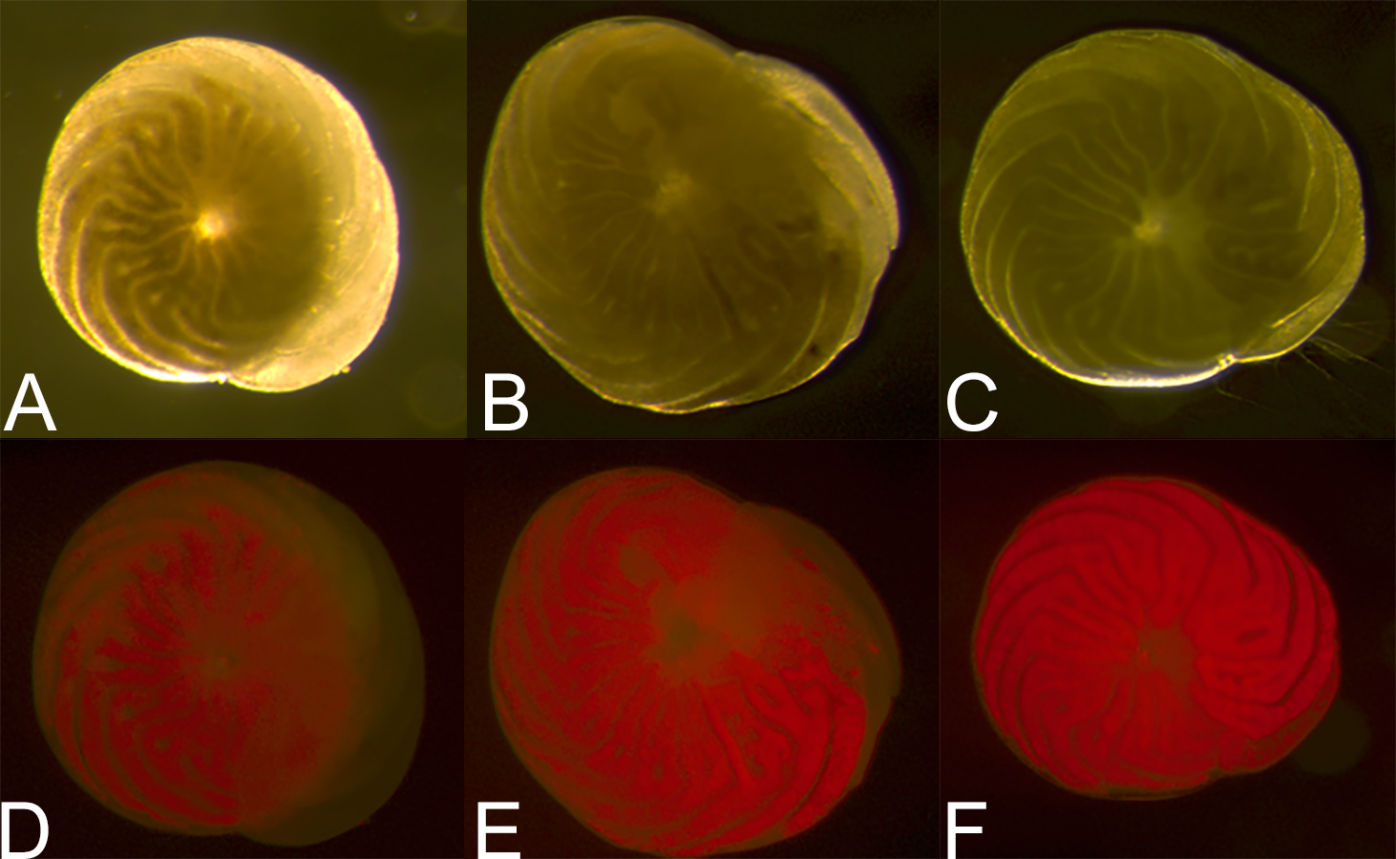
Symbiont recovery in *A. gibbosa* following 62 days in aphotic conditions. Light microscope (A–C) and fluorescent microscope (D–F) images of (A) and (D) 0 h, (B) and (E) 25 h and (C) and (F) 99 h of symbiont population recovery following 62 days in darkness (Exp. 3).

Comparing visually determined vitality with that indicated by the presence of fluorescence using a non-parametric ANCOVA test of average “live” individuals per day (either visually identified as living or by fluorescence presence) indicated a significant difference (*p* = 0.006, *df* = 32) between the measures for Experiment 1, with follow-up pairwise Mann–Whitney tests indicating significantly fewer specimens exhibited fluorescence than visible activity in the control treatment (*p* = 0.03). For the other treatments, the differences were not significant (Fig. 3). In Experiment 2, the NP-ANCOVA indicated a significant difference (*p* = 0.001, *df* = 32) in average “live” individuals per day with no recovery after PG exposure. Follow up pairwise tests showed that fluorescence indicated significantly more live individuals than visual assessment in the 3% (*p* = 0.008) and 10% (*p* = 0.008) treatments (Fig. 3). In Experiment 3, using specimens held in the dark for 62 days, a Mann–Whitney test (*df* = 19) showed no significant difference in fluorescent coverage between specimens incubated in CTG in the dark and those incubated in CTG in the light. However, specimens in the treatment incubated in the light showed significantly higher fluorescent brightness than those incubated in the dark (*p* = 0.002) (Fig. 4). Non-parametric 1-way ANOVA comparisons of specimens from the light-incubated and dark-incubated treatments of Experiment 3 to the control treatment of Experiment 1 (*df* = 67) indicated a significant difference in both brightness (*p* = 0.001) and coverage (*p* = 0.001). Follow-up pairwise comparisons showed significantly higher brightness in the control than the dark incubated specimens (*p* = 0.001), and significantly lower fluorescent coverage between control and both dark incubated (*p* = 0.006) and light incubated (*p* = 0.001) specimens. Observations of symbiont autofluorescence (Fig. 5) indicated a concentration of symbionts towards the center of the foraminifers following the extended dark treatment, with presence throughout the shell reestablished within 99 h.

**Figure 6:**
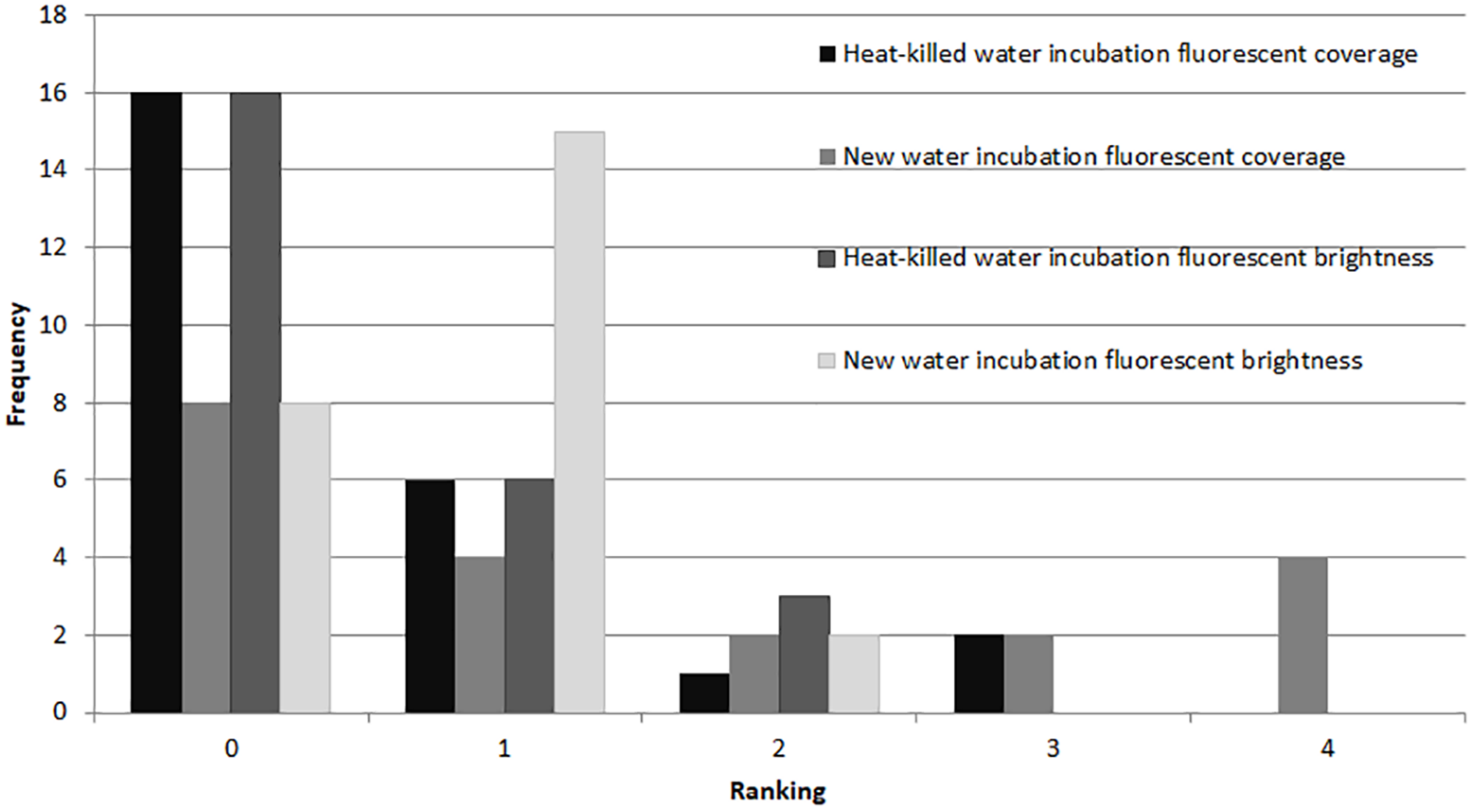
Fluorescence in heat killed *A. gibbosa*. Histogram comparing ranked fluorescent coverage and brightness in heat-killed *A. gibbosa* incubated in either the original heat-killed or new seawater (Exp. 4). Frequency includes all specimens observed.

In the microbial growth experiment (Exp. 4), a Mann–Whitney test showed no significant difference in brightness ranking (*df* = 22) between the heat-killed and new seawater treatments (Fig. 6), but the new seawater treatment showed significantly higher fluorescence coverage (*p* = 0.009).

## Discussion

### Challenges and strategies

The first goal of this research was to adapt methods utilizing CTG to determine survival of *A. gibbosa* in laboratory toxicity experiments. This objective was motivated by complications associated with dormancy that were observed during bioassay experiments (Ross & Hallock, 2014). Can fluorescence be used to quickly distinguish living from dead *A. gibbosa* in experimental studies? This goal was complicated by several factors that required preliminary experiments to find workable experimental protocols.

The primary impediment to quantitative observations of fluorescence in *A*. *gibbosa* is autofluorescence of the diatom symbionts. Hypothetically, given the consistency of the mortality pattern in previous work (Ross, 2012; Ross & Hallock, 2014), fluorescent brightness and coverage should show a consistent dose/response, since activation of CTG is linearly associated with active metabolism. However, differences in fluorescence among PG treatments were minimized by the masking effect of the symbionts (Fig. 1). These challenges prompted the use of a simple presence/absence scale of survival/mortality and the use of ranking to assess fluorescence. While this solution introduced some possibility of bias, one of the most valuable roles of foraminifers in environmental indicator work is use as a rapid, low-cost assay and bioindicator taxa. Because of the size and abundance of the shallower-dwelling species of *Amphistegina*, which occur in subtropical/tropical coastal environments nearly worldwide, qualitative measures such as visual “bleaching rank” (e.g., Hallock et al., 2006, and references therein) and visual assessment of rhizopodial activity to determine vitality (e.g., Ross & Hallock, 2014) have proven to be widely applicable. Because use of CTG requires specialized microscopy equipment, now that challenges regarding its use have been identified, further development or adaptation of these methods can include the use of quantitative image-analysis techniques, though the shape and reflective nature of the *Amphistegina* shell impose challenges to such analyses.

Suppression of CTG-induced fluorescence by PG was the second challenge that had to be solved. Solving this problem necessitated removal of experimental specimens from the treatment medium and repeated rinsing in fresh seawater before incubation in CTG. The rinsing process added another complication. Since the rate of recovery of *A. gibbosa* from chemically-induced dormancy was not known, minimizing the opportunity for specimens to recover was important to assess the presence of metabolism under dormancy, instead of during recovery. Thus, we used preliminary experiments to determine a CTG incubation period that was as short as possible, which was 30 min. Because the size of *A. gibbosa* and the short incubation period undoubtedly limited penetration by CTG, we directly exposed specimens to the 0.3 µM CTG-seawater solution in small well plates. Our CTG concentrations were higher and incubation times much shorter than previous applications (e.g., Bernhard et al., 2006; Pucci et al., 2009), where full sediment cores were incubated in 0.1 µM CTG solution for much longer periods (at least 6 h), then fixed, and CTG fluorescence was used to determine which specimens in sampled sediment layers were alive at the time of sampling. We used the preliminary experiments to adapt and employ strategies to provide consistency across our experiments and to meet our objectives, which were much different from those of previous studies.

Another challenge we encountered was photic damage associated with the high-energy excitation wavelengths required to produce fluorescence. For instance, most individuals exhibited some fluorescence following 62 days in the dark, consistent with previous observations by Smith & Hallock (1992) of recovery after 12 months in total darkness. However, experimental specimens were very sensitive to light and although activity resumed during the 72-hr recovery, symptoms of photic damage (i.e., loss of color, or “bleaching”) were subsequently observed. Photic damage during imaging also complicated observations of the individuals in the PG treatments, as these individuals also showed signs of photic stress. One of our goals was to compare results from CTG treatment with post-recovery visual assessments by observing vital activity of the foraminiferal specimens days to weeks after removal from experimental treatment. Although specimens were repeatedly examined for up to 7 days, photic damage complicated assessing the chronic influence of the chemical treatments over longer time scales. Thus, future studies with such goals will require more sets of specimens so that assessment of longer term recovery can utilize specimens that have not been previously exposed to damaging photic stress.

### Microbially-induced fluorescence

A major motivation for the use of CTG versus rose Bengal in studies that assess the assemblages of foraminifers alive in sediments when sampled is to avoid the ambiguities associated with rose Bengal. This protein stain can stain cytoplasm and microbes in dead shells (e.g., Bernhard et al., 2006). As became clear in Experiments 2 and 4, microbial growth also can produce significant fluorescence in CTG-treated dead specimens. Some fluorescence was observed in nearly half (48%) of the specimens maintained in non-sterile seawater medium for 48 hr after being heat killed (Fig. 6).

The fluorescence in the heat-killed treatments was relatively dim compared to the bright fluorescence seen in the 10% PG-exposure treatment. Many aerobic bacteria can utilize PG as a growth medium in concentrations of 10% or less (e.g., Lee et al., 2003, and references therein), and PG as a contaminant is known to be readily biodegraded by microbes (e.g., Biró et al., 2014, and references therein). In Experiment 2, if the microbial assemblage in the PG-killed foraminifers was able to bloom within the shell, feeding on the combination of PG and the degrading cytoplasm of the host and associated algae, that microbial growth could be responsible for the substantial fluorescence observed in specimens in the 10% PG treatment. Microbially-induced fluorescence may have influenced the results from other treatments; however, since all other treatments (including the 8% PG treatment in Exp. 1) showed recovery in some individuals as determined by visible activity, the influence is less clear. In future bioassay work with chemical toxicity, the influence of the chemical on microbial growth should be considered.

### Determination of mortality

A major motivation and second objective for this study was to determine if the CTG fluorescence probe could aid in distinguishing mortality from dormancy in *Amphistegina*, primarily as an aid in bioassay experiments. Other applications include determining survival potential in aphotic conditions, such as burial in sediment. Unfortunately, the results did not establish a definitive relationship between survival determined visually post recovery and esterase activity as indicated by fluorescence. Instead, many of the findings seemed counterintuitive or even contradictory, suggesting a complex relationship between stress, dormancy, survival, and fluorescence.

Even with specimens known to be dead, fluorescence ranged from none to brightest, depending upon incubation media. For example, specimens killed by treatment in deionized water (Exp. 2) showed no fluorescence, which would be expected in dead foraminifers in which there was no microbial activity. Yet heat-killed specimens (Exp. 4) kept in seawater for 48 hrs exhibited fluorescence, especially in the untreated seawater (Fig. 6). Paradoxically, fluorescence in individuals in the 48-hr 10% PG-exposure treatment with no recovery (Exp. 2) was not significantly different from controls and was significantly brighter than the 3% treatment specimens (Fig. 3), despite exposure being above the threshold previously observed to kill 100% of the experimental specimens (Ross, 2012; Ross & Hallock, 2014) and despite those individuals showing no visual evidence of survival. The highest concentration treatment in Experiment 1, 8% propylene glycol with a 72-hr recovery period, exhibited the least bright fluorescence in the experiment, and was significantly different from all other treatment concentrations.

Specimens in the 3% PG-exposure treatments provided interesting results indicating that fluorescence presence can sometimes be a better indicator of survival than visual signs. In the no-recovery treatment (Exp. 2, Fig. 3), none of the specimens exhibited visual signs of activity at the end of the 48-hr treatment, while 64% exhibited fluorescence. This percentage was similar to the ∼50% survival for the 3% PG treatment after 72-hr recovery (Exp. 1, Fig. 3). Specimens from the latter treatment were not significantly different from controls in rankings in either fluorescent coverage or brightness. In comparison, specimens in the 1.5% PG treatments with and without recovery (Exp.1 and 2) were not significantly different from control specimens.

However, specimens in the 3% PG-exposure treatment with no recovery (Exp. 2), which were likely dormant, were significantly less bright than in the other treatments (Fig. 2B), which could indicate either metabolic depression, decreased enzymatic activity, or limited uptake of media in response to PG exposure that continued during the 30 min CTG incubation. The fluorescence in some individuals was sufficiently dim to be categorized as absent, or CTG may have been excluded entirely, leading to an underestimation of survival. If the specimens in the 10% PG treatment died before they could react to the presence of PG by becoming dormant, CTG may have penetrated the shell and been taken up by the microbes throughout the dead foraminiferal specimens. This could be true in other treatments as well, leading to an overestimation of survival in treatments where mortality occurred.

An initial goal of this paper was also to compare CTG with post-recovery visual assessments to directly compare the mortality rates from each method. Unfortunately, *A. gibbosa* is light sensitive, and photic damage associated with the high-energy excitation wavelengths required to produce fluorescence, and the exposure time necessary to record it, damaged the specimens. This photic damage made visual assessment of vitality and subsequent comparisons to CTG results, unreliable.

### Behavior under dormancy

The final question motivating this study was whether use of CTG could provide insight into the behavior of *A. gibbosa* under stress, specifically in dormant states. The results offer some insights while raising more questions.

Firstly, the results suggest that dormancy suppresses metabolism and is related to survival under stress. Based on the results of the microbial-growth experiment, the differences between visual assessment of survival and presence of fluorescence (Fig. 3) becomes easier to interpret. In the control and 1.5% PG treatments, there was no or little PG to affect the foraminifers, so fluorescence presence and visually assessed survival were both near 100%. In the 3% PG treatment without recovery, the presence of fluorescence indicated much higher survival than visual assessment, though the reduction in fluorescence brightness was significant (Fig. 2B). These observations are consistent with previous observations that exposure to a 3% concentration of PG can trigger dormancy (Ross & Hallock, 2014). The 10% concentration killed the specimens, either before or in spite of any defensive reactions, and microbial growth quickly colonized the new food sources, leading to no significant difference from control specimens in the presence of fluorescence, while showing no visual signs of vitality (i.e., rhizopodial activity).

The results also suggest that recovery from dormancy can be very fast once removed from the stressor. In the darkness experiment (Exp. 3), a significant difference between the control (Exp. 1) and the dark-incubated treatment supports the hypothesis of metabolic depression in dormant individuals. The significantly higher brightness in the CTG treatment incubated in light, and its non-significant difference from the control, suggests that metabolic recovery may begin within 30 min (the length of incubation). This result suggests that any experiments involving dark-adaptation of *A. gibbosa* must be performed in darkroom conditions.

More importantly, the results of Experiment 3 indicate that recovery from darkness-induced dormancy can occur sufficiently rapidly to make metabolic depression or dormancy effective on short time scales, possibly overnight. Although research has largely focused on the photo-toxic effects of increased light levels in photosymbiotic foraminifers (i.e., Hallock, 2000), Prazeres, Uthicke & Pandolfi (2016) showed decreased antioxidant levels in *Amphistegina lobifera* exposed to low light conditions as well. Prazeres, Roberts & Pandolfi (2017) also observed that exposure to elevated temperatures and nitrate levels reduced survivorship and fecundity of *A. lobifera*, which were exacerbated by below optimal light levels. These observations demonstrate the negative impacts low- or absent light can have on LBFs, especially in warming and increasingly more nutrient-rich coastal waters, and support the hypothesis that dormancy in the absence of light may be a survival mechanism in response to physiological stress.

As ocean conditions warm, *Amphistegina* species worldwide have been observed to move poleward, and models estimate that they will continue to do so (Langer et al., 2013). Similarly, they have been found poleward of their current distributions in the geologic record, as far as 50°N and 37°S in the Miocene (Todd, 1976). Schmidt et al. (2015) found that another symbiont-bearing foraminifer, *Pararotalia* sp.*,* which has invaded the eastern Mediterranean as temperatures have warmed, demonstrates less cold-tolerance than native species. This suggests that species undergoing temperature-mediated expansion are not necessarily adapting to local conditions. If the same is true concerning light levels, the ability to enter darkness-induced dormancy on short time scales would be a valuable adaptation for tropical foraminifers expanding into areas where light availability substantially varies seasonally.

Comparing patterns of fluorescence brightness and coverage in specimens before and after a 72-hr recovery from PG exposure (Fig. 3) also provides hints of how *A. gibbosa* responds to toxic chemicals, as well as to the behavior of CTG within a foraminiferal cell. The lack of significant differences in fluorescence coverage among treatments post-recovery (Exp. 1), as well as the significant decrease in brightness and visible coverage between no-recovery (Exp. 2) and 72-hr recovery (Exp. 1) experiments, indicates that the CTG signal diminished over the recovery period. In the no-recovery experiment, CTG-induced fluorescence was largely visible in the large final chamber, in which the aperture is located and where most uptake occurs from the environment. This chamber typically houses ectoplasm that gives rise to the reticulopodia and is largely symbiont free. This chamber makes up a considerable proportion of the visible surface area, influencing estimates of fluorescence coverage. The brightness differences likely are also related to initial uptake, with most of the fluorescence concentrated in a smaller area devoid of symbionts, leading to a brighter local signal with no autofluorescent interference. The decrease in brightness over 72 h may reflect dispersal of CTG through the cytoplasm over time.

The concentration of CTG fluorescence in the outer chambers was observable in all treatments of Experiment 2, in which specimens were taken from the PG exposure, rinsed and immediately exposed to CTG. Similar concentration was not seen in the 72-hr recovery experiment (Exp. 1), the darkness experiment (Exp. 3), or the microbial growth experiment (Exp. 4). Since CTG was observed in the final chambers of the control specimens, the uptake likely reflects how foraminifers take in material from outside the shell. The fluorescence in Exp. 2 also appeared more granular than in other experiments, possibly because the CTG was still concentrated in the ectoplasm of the final chamber instead of dispersed through the endoplasm in the shell interior. The absence of concentrated fluorescence in the ectoplasm after recovery could be explained by its integration and diffusion during the recovery period. In the microbial growth experiments (Exp. 4), because the foraminiferal cell was dead, CTG entered the shell via passive diffusion. Further development of these methods may necessitate either a longer incubation in CTG, or a recovery period less than 72 hr to allow for observations of activity deeper in the cells. The question of why the fluorescence has such a granular appearance in the outer chambers is one that may be answerable by light or transmission electron microscopy. Granularity was also observed in specimens in the 10% PG treatment, where no recovery of ectoplasmic activity was observed, indicating uptake of CTG by live microbes could occur in the outer chamber.

In the 10% PG treatment (Exp. 2), specimens showed no recovery and were presumed to have died sometime during the initial PG exposure and not during post-CTG incubation. The concentration of fluorescence in the newest chamber in this treatment provides clues that the outer chamber can limit entry of toxic substances into the cell interior. That the CTG wasn’t able to diffuse more fully into the cell, despite the ectoplasm being inactive, suggests there may be a physical barrier that impedes exchange between the outer and inner chambers. This could be a matter of time, but other treatments showed more complete penetration. This hypothesis is supported by results showing the same concentration in the outer chambers of the dormant 3% PG treatment specimens, which suggests this barrier is established before metabolic activity is reduced. If present, it may be visible via TEM cytological analysis as an electron-dense body in the foramin of the penultimate chamber.

Following PG exposure and a 72-hr recovery period (Exp. 1), the 1.5% PG treatment exhibited increased brightness, while the control and 3% treatments showed similar brightness and the 8% treatment showed reduced brightness (Fig. 2A). This pattern is similar to that seen by Tzovenis et al. (2004) in a study of the effects of PG on the chlorophyte alga *Dunaliella tertiolecta*, where exposure to low concentrations led to an increase in activity (measured by growth). Tzovenis et al. (2004) suggested that PG may play the role of a micronutrient at low concentrations; the increased activity of recovered *A. gibbosa* observed here (as measured by the brightness of fluorescence) could indicate a similar effect, and support the hypothesis that the non-dormant specimens in the 1.5% treatment are actively taking in the PG in the media, or feeding on the bacteria that fed on the PG.

### Questions for further research

The results of our study raise a number of important questions about *A. gibbosa* and its reactions to stress. An obvious question raised by observations of darkness-induced dormancy is: what happens to the photosymbionts? Figure 5 shows the difference in light microscope coloration and symbiont autofluorescence in specimens of *A. gibbosa* observed at different times after removal from 62 days in aphotic conditions. Full recovery of color required approximately four days, although observations by Smith & Hallock (1992) indicate recovery can differ depending on length of time in darkness. Both imaging techniques showed a reduction in symbiont-related color, and the symbionts present when newly removed from darkness seemed to be concentrated deeper in the cell near the center, with the population dispersing throughout the cell over time. What is unclear is whether this concentration is due to movement of the symbiont population; shrinkage of the host cell; digestion of some of the total symbiont population; or a combination of the above.

As dormancy is well known in diatoms (i.e., Jewson, Rippey & Gilmore, 1981; Sicko-Goad, Stoermer & Kociolek, 1989; Itakura, Imai & Itoh, 1997; Ribeiro et al., 2011), a possible interpretation is that a small symbiont population survives, also in a dormant state, concentrated deeper in the cell when there is no light available for photosynthesis, possibly as a way for the host to safely control recovery of the symbiont population. When light becomes available, the diatoms likely asexually reproduce resulting in the observed lag between removal from darkness and recovery of healthy foraminiferal color. What is known from previous research is that the diatom-symbiont population can apparently outlive the host. When *A. gibbosa* was kept in darkness for 18 months, then reintroduced to the light, some specimens showed recovery of color, but no resumption of host vital activities (Smith & Hallock, 1992). Cytological analyses using light or transmission electron microscopy may clarify the relationship between host and symbiont in this dormant state, as well as in toxin-induced dormancy, where obvious color loss is not as easily explained. How the relationship between the host and symbionts changes on the short and long term is important to understanding how *A. gibbosa* survives environmental stress.

Another important question is: What is happening to the foraminiferal cytoplasm when dormant, either chemically or darkness induced? As discussed above, the pattern of CTG uptake observed in Experiment 2 could be explained by the presence of a barrier in the foramin between the final chamber and the penultimate chamber. If present, such a barrier might be visible via cytological analysis. Similarly, cytological analysis of individuals in long-term darkness induced dormancy can provide insight into whether the host is digesting cytoplasm or symbionts.

Most of these questions also apply to what is happening in the foraminifers when dormancy is initiated and during recovery. Cytological and metabolic studies are essential to understanding physical changes. Proteomics could also be very informative in understanding the proteins involved during initiation, dormancy, and exit from dormancy. Identifying the proteins involved might provide insight into the evolutionary origins of dormancy, and to identify the potential for dormancy in foraminiferal taxa in which it has not been directly observed.

## Conclusions

Fluorescence has the potential to be used in studies involving foraminiferal mortality, but more research is needed to confirm the relative accuracy of identification methods and to determine the ideal incubation time to allow CTG to fully enter the cell. For bioassay applications, some dormant individuals can be recognized as live without the necessity of a recovery period, but percent survival will be underestimated. In studies of live foraminiferal assemblages from stressful environments, the rapid colonization of dead shells by microbes suggests the possibility of overestimation due to the presence of recently dead individuals, while failing to identify live, dormant individuals.

By masking the signal, autofluorescence complicates the application of CTG for foraminifers that host algal endosymbionts. Because relatively little is known about the relationship between host and symbiont in stressful conditions, especially under extended darkness, other approaches such as observations of internal ultrastructure will be required. Observations of symbiont autofluorescence itself, though, may have interesting implications for a number of applications that involve visual observation of symbiont-bearing foraminifers (e.g., for signs of photic stress).

When the influence of microbial growth is considered, observed patterns of fluorescence support previous interpretations of the effect of propylene glycol on *A. gibbosa*: chronic low-level exposure at low concentrations, dormancy and metabolic depression above a certain threshold, and mortality at higher concentrations. These patterns also support the hypothesis of some kind of barrier between the outer and inner chambers, such as the formation of an “apertural plug”.

##  Supplemental Information

10.7717/peerj.5304/supp-1 Supplemental Information 1 1–13 PG%- no recovery experiment- raw data for Experiment 2, exposure to propylene glycol and CellTracker Green fluorescence with no recovery period

10.7717/peerj.5304/supp-2 Supplemental Information 2 Microbial growth in dead foraminifera - raw data for experiment observing microbial growth, as indicated by CellTracker Green, in heat-killed foraminifera

10.7717/peerj.5304/supp-3 Supplemental Information 3 12–24 PG% Fluorescence Experiment- raw data for Experiment 1, exposure to propylene glycol and CellTracker Green fluorescence after a 72 hr recovery period

10.7717/peerj.5304/supp-4 Supplemental Information 4 Comparison between treatments- raw data of comparative statistics between experiments and initial figure designs

## Additional Information and Declarations

### Field Study Permissions

The following information was supplied relating to field study approvals (i.e., approving body and any reference numbers): Foraminiferal samples were collected under the Florida Keys National Marine Sanctuary Permit FKNMS-2015-026.

### Data Availability

The following information was supplied regarding data availability: The raw data are provided in the Supplemental Files.
